# Safety of Short‐Term Trimethoprim–Sulfamethoxazole Use for Uncomplicated Cystitis: A Nationwide Retrospective Cohort Study

**DOI:** 10.1002/pds.70342

**Published:** 2026-03-02

**Authors:** Jumpei Taniguchi, Shotaro Aso, Hideo Yasunaga

**Affiliations:** ^1^ Department of Clinical Epidemiology and Health Economics, School of Public Health The University of Tokyo Tokyo Japan; ^2^ Department of Health Services Research, Graduate School of Medicine The University of Tokyo Tokyo Japan

**Keywords:** adverse reaction, Asia, cystitis, drug hypersensitivity, trimethoprim‐sulfamethoxazole

## Abstract

**Background:**

Trimethoprim–sulfamethoxazole (TMP–SMX) is associated with hypersensitivity and other adverse reactions. East Asian–specific genetic susceptibility may increase severe adverse events, limiting the therapeutic use of TMP–SMX for uncomplicated cystitis in Japan. However, data on its short‐term safety in this population are scarce. This study compared the short‐term safety of TMP–SMX with fluoroquinolones for the treatment of uncomplicated cystitis using a nationwide Japanese claims database.

**Methods:**

We conducted a retrospective cohort study using the JMDC Claims Database (2006–2022). We included female outpatients aged ≥ 18 years with acute uncomplicated cystitis who were newly treated with TMP–SMX or fluoroquinolones (levofloxacin, ciprofloxacin, or tosufloxacin). The primary outcome was drug‐induced hypersensitivity (anaphylaxis or treatment‐requiring rash) within 7 days after the prescription of these drugs. The secondary outcomes included treatment failure, all‐cause hospitalization, and other adverse events. Propensity score‐overlap weighting was applied to adjust for confounding. Subgroup analyses were stratified by age (< 50 or ≥ 50 years).

**Results:**

Among 50 773 eligible patients (TMP–SMX, 2.1%; fluoroquinolones, 97.9%), the baseline characteristics were well balanced after weighting. The incidence of hypersensitivity did not differ significantly between the groups (0.9% vs. 0.7%; risk difference, 0.3%; 95% confidence interval, −0.3% to 0.8%; *p* = 0.410). No significant differences were observed for secondary outcomes. Subgroup analyses showed consistent results.

**Conclusions:**

Short‐term use of TMP–SMX was not associated with increased risks of adverse events or treatment failure compared with fluoroquinolones. TMP–SMX may represent a reasonable first‐line option for acute uncomplicated cystitis in Japan.

## Key Points

- East Asian–specific genetic susceptibility may increase the risk of adverse reactions to trimethoprim–sulfamethoxazole (TMP–SMX), potentially limiting its therapeutic use in this population.
- In a nationwide Japanese claims database, TMP–SMX use for acute uncomplicated cystitis was not associated with increased adverse events or treatment failure compared with fluoroquinolones.
- TMP–SMX represents a reasonable first‐line option for acute uncomplicated cystitis in Japan.

## Plain Language Summary

Uncomplicated cystitis is a common bladder infection that is usually treated with short courses of oral antibiotics. A medication called trimethoprim–sulfamethoxazole (TMP–SMX) is recommended as a first‐line treatment in many countries, but its use is limited in Japan and other East Asian regions because certain genetic traits in these populations may increase the risk of severe allergic reactions. As a result, fluoroquinolones are used more often, despite concerns that their overuse contributes to antibiotic resistance. In this study, we used a large nationwide health insurance database in Japan to evaluate whether TMP–SMX is safe for short‐term use in women with uncomplicated cystitis. We examined more than 50 000 treatment episodes and compared patients who received TMP–SMX with those who received fluoroquinolones. We found that adverse reactions were uncommon and occurred at similar rates in both groups. These findings were consistent across age groups and additional analyses. Overall, our results suggest that TMP–SMX is effective and provides similar short‐term outcomes to fluoroquinolones in the treatment of uncomplicated cystitis in Japanese women, and may serve as a reasonable first‐line option while supporting efforts to reduce unnecessary fluoroquinolone use.

**Abbreviations**
CI: confidence interval
ICD‐10: International Classification of Diseases 10th revision
RD: risk difference
TMP–SMX: trimethoprim–sulfamethoxazole
WHO‐ATC: World Health Organization Anatomical Therapeutic Chemical

## 1 Introduction

Trimethoprim–sulfamethoxazole (TMP–SMX) is an oral antibiotic combination with action against major uropathogens, including *Escherichia coli* . A guideline devised by the Infectious Diseases Society of America and the European Society for Microbiology and Infectious Diseases recommends TMP–SMX as first‐line therapy for uncomplicated cystitis, alongside nitrofurantoin, trimethoprim alone, and pivmecillinam, provided that local resistance rates do not exceed 20% [1, 2, 3].

In Japan and several other East Asian countries, nitrofurantoin, trimethoprim alone, and pivmecillinam are not commercially available. Consequently, fluoroquinolones are often prescribed owing to their accessibility, broad antimicrobial spectrum, and favorable safety profile [4, 5, 6, 7, 8, 9]. However, the widespread use of fluoroquinolones has contributed to a steady rise in *E. coli* resistance [4, 5, 6, 7, 8, 9, 10, 11, 12], raising public health concerns regarding antimicrobial stewardship. Therefore, fluoroquinolones should be avoided, except for complicated cases or when first‐line agents are unsuitable [3]. TMP–SMX is widely available and effective against common uropathogens, constituting a reasonable narrow‐spectrum alternative to fluoroquinolones for the treatment of uncomplicated cystitis [3]. Nevertheless, TMP–SMX has been reported to be associated with a higher risk of adverse drug reactions—such as hypersensitivity, gastrointestinal symptoms, renal dysfunction, and electrolyte abnormalities—compared with other antibiotics [13, 14, 15, 16, 17]. Moreover, several East Asian–specific human leukocyte antigen (HLA) alleles have been identified as risk factors for sulfonamide‐induced severe cutaneous reactions, leading to concerns that TMP–SMX–related serious adverse events may be more frequent in this population and thereby contributing to its limited clinical use [7, 9, 10, 18, 19, 20, 21]. Despite these concerns, evidence regarding the safety of short‐term TMP–SMX for acute uncomplicated cystitis remains limited in East Asian populations [14, 22, 23].

Therefore, this study aimed to evaluate the short‐term safety of TMP–SMX compared with fluoroquinolones for the treatment of acute uncomplicated cystitis in Japan using a large‐scale claims database to determine whether TMP–SMX could serve as a safe alternative to fluoroquinolones in real‐world clinical practice.

## 2 Methods

### 2.1 Data Source and Study Design

This retrospective cohort study analyzed data extracted from the nationwide health insurance JMDC Claims Database (JMDC Inc., Tokyo, Japan), which includes anonymised information on more than seven million beneficiaries [24, 25, 26]. The database collects claims submitted by hospitals, clinics, and pharmacies and mainly covers employees of medium‐to‐large companies and their family members [25]. Consequently, the dataset is skewed toward individuals younger than 65 years of age and does not include those aged 75 years or above [25]. The JMDC consolidates information on patient demographics (e.g., age and sex), dates of medical encounters, diagnoses recorded using the International Classification of Diseases, 10th Revision (ICD‐10) codes, prescriptions classified according to the World Health Organization Anatomical Therapeutic Chemical (WHO‐ATC) system, and procedure records [25]. The JMDC database has been extensively used in clinical and epidemiological research in Japan [26].

The present study analyzed claims data from January 2006 through May 2022. Ethical approval was obtained from the Institutional Review Board of the University of Tokyo [approval number: 10862–(1)]. The requirement for informed consent was waived because the database comprises de‐identified secondary data.

### 2.2 Patient Selection

We identified female outpatients aged ≥ 18 years diagnosed with acute cystitis (ICD‐10 code N30.0) who were newly treated with TMP–SMX or fluoroquinolones (levofloxacin, ciprofloxacin, or tosufloxacin) between January 2006 and March 2022. The index date was defined as the date of the first eligible prescription. Patients who received a prescription for at least 3 days were included.

The exclusion criteria were as follows: (i) complicated cystitis, (ii) patients diagnosed with pyelonephritis on the index date, (iii) concomitant prescription of both study drugs on the index date, (iv) concomitant prescription of oral antibiotics other than the study drugs on the index date, (v) administration of intravenous antibiotics on the index date, (vi) prescribed duration of the study drug exceeded 7 days, (vii) history of urinary tract infection (cystitis or pyelonephritis) within 6 months from the index date, (viii) patients without a six‐month look‐back period before the index date, and (ix) pregnancy. Patients who initiated a new study drug in a different calendar year were eligible for re‐inclusion in the cohort for that year. Complicated cystitis was defined as the presence or diagnostic history of any of the following during the look‐back period: structural or functional abnormalities of the urinary tract (e.g., urolithiasis, obstruction/hydronephrosis, neurogenic bladder or voiding dysfunction, or congenital anomalies); urological devices or procedures (e.g., indwelling or intermittent catheterization, ureteral stent placement, urinary diversion, bladder irrigation, or urologic surgery); or an immunocompromised state (e.g., diabetes mellitus, autoimmune disease, human immunodeficiency virus infection, chronic kidney disease, malignancy, or use of systemic corticosteroids, immunosuppressants, or antineoplastic agents). The ICD‐10 and WHO‐ATC codes used to determine the exclusion criteria are presented in Table S1.

### 2.3 Exposure

Patients were categorized into two groups according to the prescribed antibiotics: the TMP–SMX group and the fluoroquinolone group (levofloxacin, ciprofloxacin, or tosufloxacin).

### 2.4 Covariates

A six‐month baseline period was used to ascertain eligibility and identify the covariates. We extracted the following baseline covariates from the database. Patient factors included age, year, and duration of prescription (3, 4, 5, 6, or 7 days). Diagnostic tests on the index date comprised urinalysis, Gram staining, urine culture, ultrasonography, imaging examinations (X‐ray or computed tomography), and blood tests. Comorbidities assessed included allergic diseases (allergic rhinitis, atopic dermatitis, asthma, and urticaria), hypertension, dyslipidemia, cerebrovascular disease, neuromuscular disorders, cardiovascular disease, liver disease, osteoporosis, osteoarthritis, gynecological disorders, dementia, mood disorders, and schizophrenia. Comorbidities were assessed using diagnoses recorded during the 6‐month look‐back period. Concomitant medications were classified according to the assessment window. Analgesics and antipyretics (paracetamol and non‐steroidal anti‐inflammatory drugs) and Japanese herbal medicines (Kampo), which primarily reflect acute disease severity, were assessed on the index date only. In contrast, diuretics, hormone replacement therapy, psychotropic medications, and proton pump inhibitors were assessed within the 3 months prior to the index date to capture current medication exposure, consistent with prescribing practices in Japan. Covariates were selected based on their potential clinical associations with disease severity, antibiotic selection, and outcomes [27, 28, 29, 30]. Because body size‐related variables, such as body weight and body mass index, were not available in our database, we adjusted for a broad range of comorbidities commonly associated with obesity and related lifestyle factors as proxy measures of body size‐related characteristics. The corresponding ICD‐10 and WHO‐ATC codes for these covariates and medications are listed in Table S2.

### 2.5 Outcomes

The primary outcome was drug‐induced hypersensitivity reactions occurring within 7 days after the index date, based on ICD‐10 codes in combination with treatment information. This composite outcome included diagnoses of anaphylaxis with epinephrine administration and cutaneous adverse reactions. Cutaneous adverse reactions were defined as Stevens–Johnson syndrome/toxic epidermal necrolysis, and drug‐treated rash requiring systemic corticosteroids (oral or intravenous) or a combination of antihistamines and topical corticosteroids. The secondary outcomes included treatment failure, all‐cause hospitalization, and other adverse events, occurring within 7 days after the index date. Treatment failure was defined as a diagnosis of pyelonephritis, or additional prescription or use of oral/intravenous antibiotics. Other adverse events included gastrointestinal symptoms, electrolyte abnormalities, hypoglycemia, and acute renal failure. In addition, we reported the occurrence of serious arrhythmias and all‐cause mortality within 7 days after the index date. Detailed definitions of the ICD‐10 codes, ATC codes, and treatment criteria for each outcome are provided in Table S3.

The overall study design and assessment timeline are summarized in Figure S1.

### 2.6 Statistical Analysis

We conducted a propensity score overlap weighting analysis to compare outcomes between the TMP–SMX and fluoroquinolone groups [31, 32, 33]. Propensity scores were estimated using logistic regression, modeling assignment to the TMP–SMX group as the dependent variable with baseline covariates as independent variables. Under the overlap weighting scheme, each patient is assigned a weight based on the probability of receiving the opposite treatment group. Thus, patients treated with TMP–SMX were weighted by 1 minus the propensity score, and those treated with fluoroquinolones were weighted by the propensity score. This approach places greater emphasis on patients with overlapping treatment probabilities, thereby improving covariate balance and reducing the influence of extrapolation [31, 32, 33]. Balance between groups was assessed using standardized mean differences, with absolute values below 10% indicating acceptable balance [34]. Risk differences (RDs) and 95% confidence intervals (CIs) were estimated using binomial regression models with propensity score overlap weighting. To account for repeated observations of the same individuals across the years, all weighted analyses were performed using robust standard errors clustered at the patient level.

### 2.7 Subgroup and Sensitivity Analyses

We performed subgroup analyses stratified by age (< 50 or ≥ 50 years) to compare the treatment effects of the study drugs before and after menopausal transition [3, 11, 35].

Moreover, we conducted sensitivity analyses by restricting the sample to (i) patients who received a 3‐day antibiotic course because this is generally sufficient for uncomplicated cystitis [3]; (ii) patients who received TMP–SMX at a trimethoprim‐equivalent dose of 320 mg/day [160/800 mg (1 double‐strength tablet) twice daily], consistent with the guideline‐recommended regimen [3]; (iii) patients who were included only at their first cohort entry, without duplicate inclusion, to account for the potential influence of prior cystitis treatment history on antibiotic selection; and (iv) patients diagnosed with anaphylaxis based on diagnostic codes alone to account for possible cases in which epinephrine may not have been administered. In addition, we performed sensitivity analyses using inverse probability of treatment weighting to estimate the average treatment effect on the untreated, thereby assessing the expected effect of TMP–SMX among patients who received fluoroquinolones.

For all subgroup and sensitivity analyses, propensity scores and overlap weights were estimated after applying each patient restriction.

Continuous variables were compared using *t*‐tests and expressed as means with standard deviations (SD). Categorical variables were compared using the chi‐squared test and reported as frequencies with percentages. Two‐tailed *p* values < 0.05 were considered statistically significant. All analyses were conducted using STATA/SE (version 19.5; StataCorp, College Station, TX, USA).

## 3 Results

**FIGURE 1:**
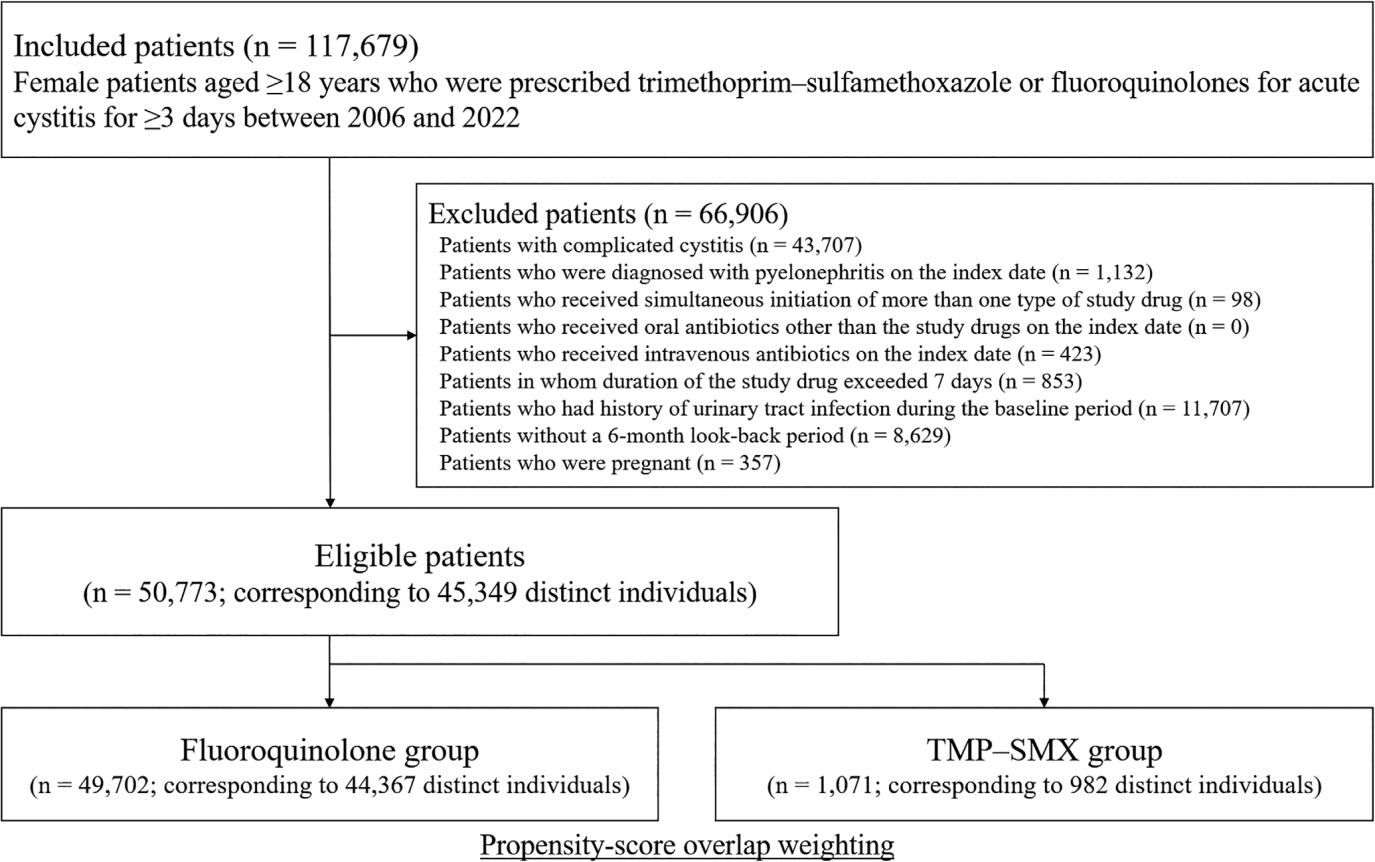
Study design.

We identified 117 679 patients who received study antibiotics for acute cystitis between January 2006 and May 2022 (Figure 1). A total of 50 773 patients were eligible, corresponding to 45 349 distinct individuals. Overall, 2.1% (1071/50 773) of patients received TMP–SMX and 97.9% (49 702/50 773) of patients received fluoroquinolones. These corresponded to 982 and 44 367 distinct individuals in the TMP–SMX and fluoroquinolone groups, respectively. In the fluoroquinolone group, 91.0% of patients received levofloxacin, 3.3% received ciprofloxacin, and 5.7% received tosufloxacin. The mean daily dose was 308.7 mg (SD, 42.1) in the TMP–SMX group (trimethoprim‐equivalent), and 492.7 mg (SD, 64.9) for levofloxacin, 523.2 mg (SD, 98.5) for ciprofloxacin, and 413.7 mg (SD, 69.2) for tosufloxacin in the fluoroquinolone group.

Table 1 presents the baseline patient characteristics. The mean age was approximately 42 years in both groups. Urinalysis was performed in approximately 90% of patients, Gram staining for approximately 10%, and urine culture for approximately 35% of patients in both groups. In the unadjusted population, no cases of anaphylaxis or Stevens–Johnson syndrome/toxic epidermal necrolysis occurred in the TMP–SMX group, whereas one case of Stevens–Johnson syndrome/toxic epidermal necrolysis was observed in the fluoroquinolone group. There was no significant difference in the primary outcome between the two groups (0.9% vs. 0.6%; RD, 0.3%; 95% CI, −0.3% to 0.9%). There were also no significant differences in any of the secondary outcomes. No serious arrhythmias or all‐cause deaths were observed in either group.

**TABLE 1:** Patient characteristics.

Variables	Unweighted			Propensity score‐overlap weighted		
	Fluoroquinolone (*n* = 49 702)	TMP–SMX (*n* = 1071)	Standardized difference	Fluoroquinolone	TMP–SMX	Standardized difference
Age, years, mean (SD)	41.8 (13.7)	42.6 (13.8)	5.8	42.6 (13.7)	42.6 (13.8)	0.0
Year, *n* (%)						
2006–2015	8495 (17.1)	127 (11.9)	−14.9	(12.0)	(12.0)	0.0
2016–2022	41 207 (82.9)	944 (88.1)	14.9	(88.0)	(88.0)	0.0
Duration of prescription, days, *n* (%)						
3	7904 (15.9)	544 (50.8)	79.7	(49.3)	(49.3)	0.0
4	6441 (13.0)	87 (8.1)	−15.8	(8.4)	(8.4)	0.0
5	24 591 (49.5)	327 (30.5)	−39.4	(31.5)	(31.5)	0.0
6	871 (1.8)	5 (0.5)	−12.3	(0.5)	(0.5)	0.0
7	9895 (19.9)	108 (10.1)	−27.8	(10.4)	(10.4)	0.0
Diagnostic tests, *n* (%)						
Urinalysis	43 808 (88.1)	930 (86.8)	−3.9	(87.0)	(87.0)	0.0
Gram staining	4641 (9.3)	149 (13.9)	14.3	(13.7)	(13.7)	0.0
Urine culture	18 381 (37.0)	356 (33.2)	−7.8	(33.4)	(33.4)	0.0
Ultrasonography	3209 (6.5)	112 (10.5)	14.4	(10.3)	(10.3)	0.0
X‐ray	551 (1.1)	16 (1.5)	3.4	(1.4)	(1.4)	0.0
CT	350 (0.7)	20 (1.9)	10.3	(1.7)	(1.7)	0.0
Blood tests	2000 (4.0)	68 (6.3)	10.5	(6.1)	(6.1)	0.0
Comorbidities, *n* (%)						
Allergic diseases						
Allergic rhinitis	10 249 (20.6)	222 (20.7)	0.3	(20.7)	(20.7)	0.0
Atopic dermatitis	1745 (3.5)	38 (3.5)	0.2	(3.5)	(3.5)	0.0
Asthma	3449 (6.9)	59 (5.5)	−5.9	(5.6)	(5.6)	0.0
Urticaria	1549 (3.1)	27 (2.5)	−3.6	(2.6)	(2.6)	0.0
Hypertension	3109 (6.3)	62 (5.8)	−2.0	(5.8)	(5.8)	0.0
Dyslipidemia	3704 (7.5)	80 (7.5)	0.1	(7.5)	(7.5)	0.0
Cerebrovascular disease	528 (1.1)	12 (1.1)	0.6	(1.1)	(1.1)	0.0
Neuromuscular disorders	263 (0.5)	8 (0.7)	2.7	(0.7)	(0.7)	0.0
Cardiovascular disease	610 (1.2)	7 (0.7)	−5.9	(0.7)	(0.7)	0.0
Liver disease	1267 (2.5)	32 (3.0)	2.7	(3.0)	(3.0)	0.0
Osteoporosis	789 (1.6)	21 (2.0)	2.8	(1.9)	(1.9)	0.0
Osteoarthritis	1666 (3.4)	48 (4.5)	5.8	(4.4)	(4.4)	0.0
Gynecological disorders	5825 (11.7)	129 (12.0)	1.0	(12.0)	(12.0)	0.0
Dementia	18 (0.0)	0 (0.0)	−2.7	(0.0)	(0.0)	0.0
Mood disorders	1917 (3.9)	42 (3.9)	0.3	(3.9)	(3.9)	0.0
Schizophrenia	523 (1.1)	15 (1.4)	3.2	(1.4)	(1.4)	0.0
Medications, *n* (%)						
Paracetamol	2037 (4.1)	64 (6.0)	8.6	(5.7)	(5.7)	0.0
NSAIDs	3107 (6.3)	49 (4.6)	−7.4	(4.6)	(4.6)	0.0
Herbal medicines	2925 (5.9)	41 (3.8)	−9.6	(3.9)	(3.9)	0.0
Diuretics	222 (0.4)	4 (0.4)	−1.1	(0.4)	(0.4)	0.0
Hormone replacement therapy	1516 (3.1)	43 (4.0)	5.2	(3.9)	(3.9)	0.0
Psychotropic medications	2335 (4.7)	59 (5.5)	3.7	(5.4)	(5.4)	0.0
Proton pump inhibitors	1534 (3.1%)	37 (3.5%)	2.1	(3.4)	(3.4)	0.0

*Note:* The total number of etiologies does not add up to 100% as more than one cause can be assigned to a single patient.

Abbreviations: CT, computed tomography; NSAIDs, non‐steroidal anti‐inflammatory drugs; TMP–SMX, trimethoprim–sulfamethoxazole.

### 3.1 Main Analysis

After propensity‐score overlap weighting, all baseline characteristics were well‐balanced between the groups (Table 1). Additional balance diagnostics are presented in Figure S2. In the propensity‐score analysis, there was no significant difference in the primary outcome (0.9% vs. 0.7%; RD, 0.3%; 95% CI, −0.3% to 0.8%) between the two groups (Table 2). There were no significant differences in any of the secondary outcomes, including treatment failure.

**TABLE 2:** Comparison of outcomes between the propensity score weighted groups.

	Fluoroquinolone	TMP–SMX	Risk difference	95% confidence interval	*p*
Primary outcome (%)					
Drug‐induced hypersensitivity reactions	0.7 (0.6)	0.9 (0.9)	0.3	−0.3 to 0.8	0.410
Anaphylaxis	0.0 (0.0)	0.0 (0.0)	N/A	N/A	N/A
Stevens–Johnson syndrome/toxic epidermal necrolysis	0.0 (0.0)	0.0 (0.0)	N/A	N/A	N/A
Rash requiring pharmacotherapy	0.7 (0.6)	0.9 (0.9)	0.3	−0.3 to 0.8	0.410
Secondary outcomes (%)					
Treatment failure	11.3 (10.6)	11.3 (11.4)	0.0	−2.0 to 2.0	0.989
All‐cause hospitalization	0.2 (0.2)	0.3 (0.3)	0.1	−0.3 to 0.4	0.754
Other adverse events	0.3 (0.2)	0.2 (0.2)	−0.1	−0.3 to 0.2	0.614
Gastrointestinal symptoms	0.2 (0.2)	0.1 (0.1)	−0.1	−0.3 to 0.1	0.163
Electrolyte abnormalities	0.0 (0.0)	0.1 (0.1)	0.1	−0.1 to 0.2	0.389
Hypoglycemia	0.0 (0.0)	0.0 (0.0)	N/A	N/A	N/A
Acute renal failure	0.0 (0.0)	0.0 (0.0)	N/A	N/A	N/A

*Note:* Values in parentheses indicate the crude outcomes.

Abbreviation: TMP–SMX, trimethoprim–sulfamethoxazole.

### 3.2 Subgroup and Sensitivity Analyses

**FIGURE 2:**
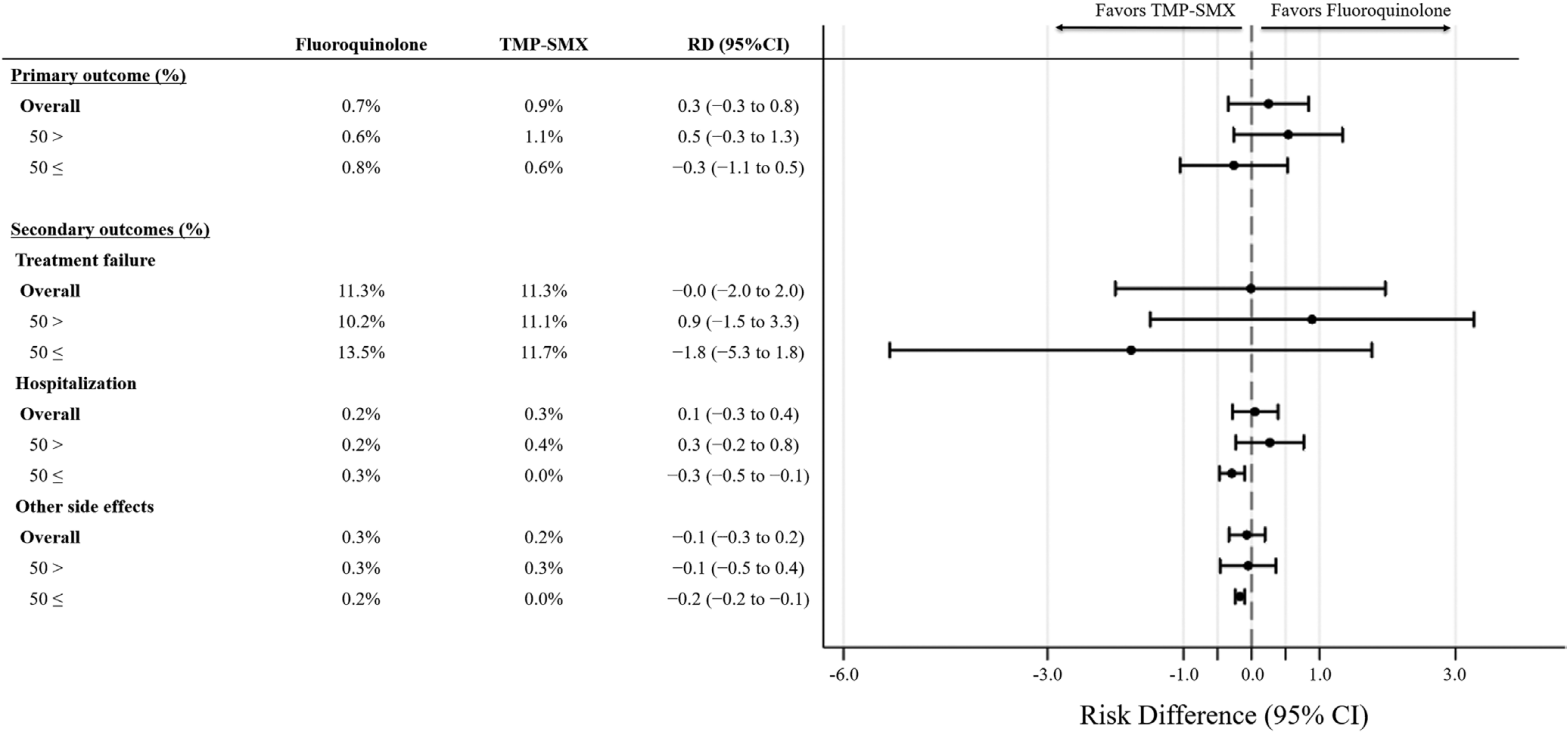
Subgroup analysis between the propensity score weighted groups stratified by age (< 50 or ≥ 50 years).

The results of the subgroup analysis are presented in Figure 2. In analyses stratified by age (< 50 and ≥ 50 years), the primary and secondary outcomes were generally consistent with the main findings.

The results of the sensitivity analyses were largely consistent with those of the main analysis (Table S4).

## 4 Discussion

In this nationwide cohort study, we compared clinical outcomes between TMP–SMX and fluoroquinolones in female patients with uncomplicated cystitis. There was no significant difference in adverse events or treatment failure between short‐term TMP–SMX and fluoroquinolone use. These findings suggest that TMP–SMX may represent a reasonable alternative to fluoroquinolones for uncomplicated cystitis in countries like Japan, where other narrow‐spectrum options are limited.

A major strength of this study is the use of a nationwide claims database in Japan, which comprehensively captured real‐world prescription patterns and outcomes across diverse clinical settings. Although several randomized controlled trials compared antibiotics including TMP–SMX for uncomplicated cystitis, most were conducted during the 1980s–1990s in Western populations, with limited inclusion of Asian participants [14, 36]. A randomized controlled trial from South Korea in 2007 that compared ciprofloxacin and TMP–SMX could not evaluate safety outcomes because the sample size was only 75 [37]. Because previous retrospective pharmacovigilance and observational studies did not adjust for confounding factors, the short‐term safety of TMP–SMX for uncomplicated cystitis in Asian populations could not be clarified [13, 14, 20]. In contrast, our study included more than 50 000 episodes of uncomplicated cystitis among women in Japan and adjusted for confounding related to disease severity and treatment indication. We were able to specifically assess the short‐term safety of TMP–SMX in the clinical context where it would most likely be considered, focusing on outpatient prescriptions with short treatment durations. To the best of our knowledge, this is the first study to evaluate the short‐term safety of TMP–SMX in an East Asian population.

TMP–SMX is an appropriate antibiotic for uncomplicated cystitis because of its narrow spectrum and activity against common uropathogens [3]. However, its use in Japan has been limited by concerns over sulfonamide‐induced hypersensitivity reactions, particularly in individuals carrying East Asian–specific HLA alleles such as HLA‐B*13:01, HLA‐B*15:02, and HLA‐A*11:01 [18, 21]. The use of TMP–SMX has been appropriate for treating uncomplicated cystitis in Japan because of the low resistance rate of *E. coli* (approximately 10%) [38, 39]. Nevertheless, fluoroquinolones were prescribed in more than half of cases with uncomplicated cystitis, whereas TMP–SMX was used in less than 1%, suggesting that clinicians continue to avoid TMP–SMX even for short‐course therapy [8]. Our results indicate that short‐term TMP–SMX therapy was not associated with an increased risk of adverse events compared with fluoroquinolones. Overall, these findings support TMP–SMX as a reasonable treatment option for uncomplicated cystitis, even among Japanese women.

Our findings are generally consistent with a previous Cochrane review summarizing randomized controlled trials, which also found no difference in efficacy or safety between fluoroquinolones and TMP–SMX for uncomplicated cystitis in women [36]. Although prior studies suggested a higher risk of rash with TMP–SMX, no such difference was observed in our cohort. Exanthematous drug eruptions, one of the most common adverse reactions associated with TMP–SMX, typically occur 4–14 days after treatment initiation; therefore, the relatively short, guideline‐recommended treatment duration and the strict definition of rash requiring treatment in our study may explain these findings [14, 36, 40, 41]. While our results support the short‐term safety of TMP–SMX for uncomplicated cystitis, antibiotic selection should still be individualized based on patient age, ethnicity, comorbidities, concomitant medications, and personal or family history of allergy, as well as local antibiograms.

This study has several limitations. First, despite propensity score adjustment, residual confounding may have persisted due to unmeasured factors such as allergy history, bacterial data, symptom severity, or physicians' prescribing preferences. Although sensitivity analyses supported the robustness of our findings, confounding by indication remains a potential source of bias. Second, our database primarily includes working‐age individuals, which may limit the generalizability of our findings to older populations or patients with specific comorbidities. In addition, because TMP–SMX and fluoroquinolones may be avoided in younger women of childbearing potential in routine clinical practice, restriction to these agents may further limit generalizability to the broader female population. Third, the small number of patients treated with TMP–SMX and the rarity of hypersensitivity events limited the statistical power to detect small between‐group differences. Moreover, because anaphylaxis and Stevens–Johnson syndrome/toxic epidermal necrolysis were extremely rare, the main analysis primarily captured rashes requiring pharmacological treatment. Therefore, although our findings suggest that serious adverse events were uncommon, they do not fully exclude the possibility of rare but clinically important risks associated with TMP–SMX use. Finally, misclassification of diagnoses and outcomes due to coding inaccuracies is possible; however, the use of combined diagnostic and treatment criteria likely improved specificity.

## 5 Conclusion

This large, real‐world study suggests that TMP–SMX and fluoroquinolones have similar short‐term outcomes in the treatment of acute uncomplicated cystitis among Japanese women. Considering the narrow antimicrobial spectrum of TMP–SMX and potential to reduce fluoroquinolone overuse, TMP–SMX may represent a reasonable first‐line option for uncomplicated cystitis.

## Author Contributions

J.T. conceived, designed, analyzed, and coordinated the study. J.T. and S.A. wrote the first draft of the manuscript. H.Y. contributed to data interpretation and assisted with manuscript preparation. S.A. and H.Y. critically reviewed the manuscript. H.Y. critically appraised the manuscript. All the authors have read and approved the final version of this manuscript.

## Funding

The authors have nothing to report.

## Ethics Statement

This study was approved by the Institutional Review Board of the University of Tokyo. The board waived the need for informed consent as this was a retrospective study.

## Consent

The Institutional Review Board waived the requirement for informed consent because of the anonymous nature of the data.

## Conflicts of Interest

The authors declare no conflicts of interest.

## Supporting information


**Figure S1:** Graphical description of the study design.
**Figure S2:** Propensity score distributions before and after overlap weighting and effective sample size.
**Table S1:** ICD‐10 and ATC codes used for exclusion criteria.
**Table S2:** ICD‐10 and ATC codes used for covariates.
**Table S3:** ICD‐10 and ATC codes for outcome definitions.
**Table S4:** Comparison of outcomes between propensity score weighted groups in the sensitivity analyses.

## Data Availability

The datasets used in this study are not available.
