# Supplementary material for: Safety of Short‐Term Trimethoprim–Sulfamethoxazole Use for Uncomplicated Cystitis: A Nationwide Retrospective Cohort Study
Source: Pharmacoepidemiol Drug Saf. 2026 Mar 2;35(3):e70342. doi: 10.1002/pds.70342 (PMC12954357; doi:10.1002/pds.70342)
Supplement: Supplementary file 1 — Figure S1: Graphical description of the study design. Figure S2: Propensity score distributions before and after overlap weighting and effective sample size. Table S1: ICD‐10 and ATC codes used for exclusion criteria. Table S2: ICD‐10 and ATC codes used for covariates. Table S3: ICD‐10 and ATC codes for outcome definitions. Table S4: Comparison of outcomes between propensity score weighted groups in the sensitivity analyses. [file PDS-35-e70342-s001.docx]

**Supplementary Figure 1. Graphical description of the study design.**

**
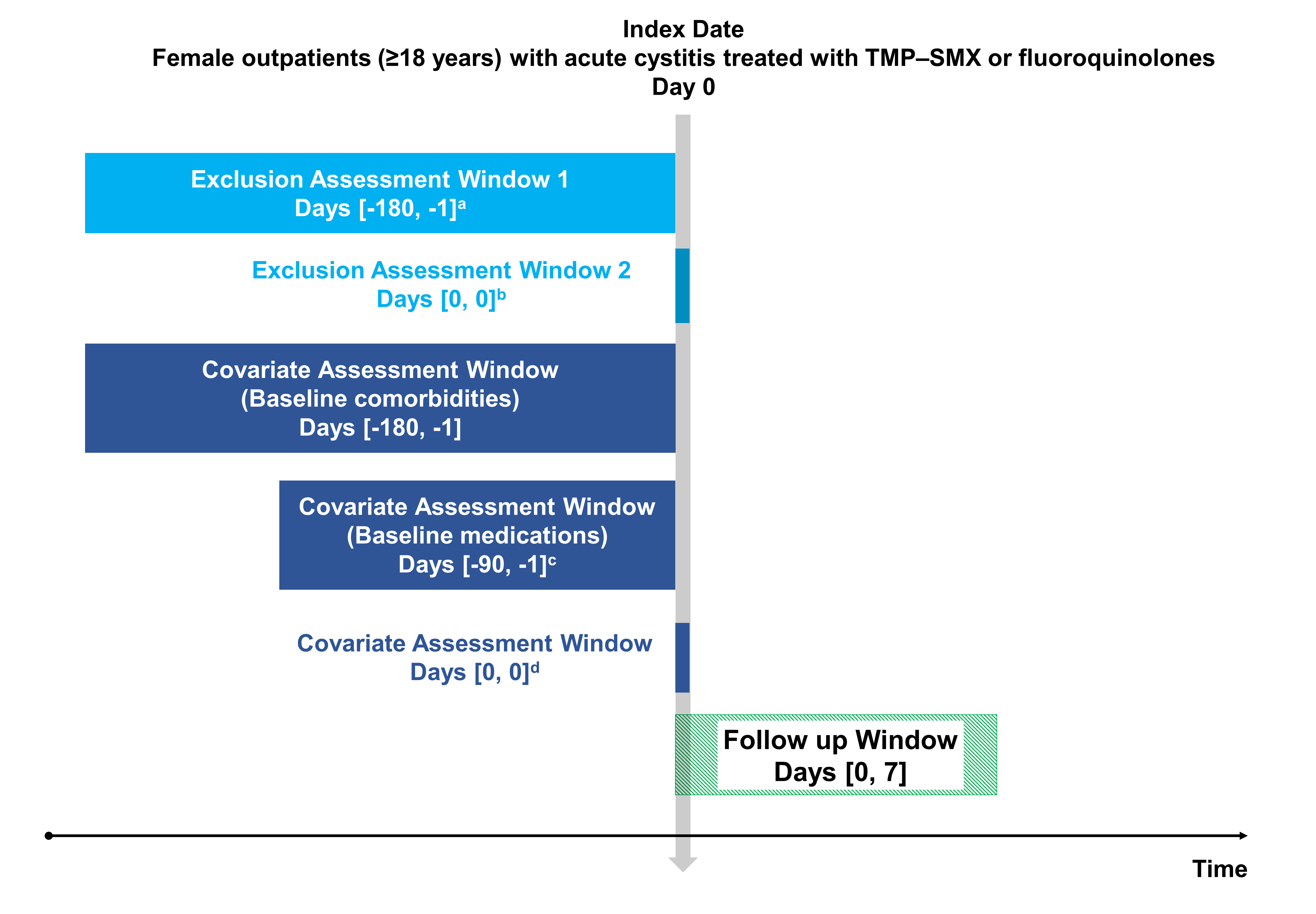
**

Different assessment windows were applied to the following variables: (a) prior history associated with complicated cystitis, urinary tract infections, or pregnancy; (b) diagnosis of pyelonephritis, use of other or multiple antibiotics, or antibiotic prescription duration >7 days; (c) prior use of diuretics, hormone replacement therapy, psychotropic medications, or proton pump inhibitors; and (d) baseline covariates (age and calendar year), prescriptions of comcomittant medications (paracetamol, NSAIDs, or Kampo medicines), and orders for diagnostic tests.

**Supplementary Figure 2. Propensity score distributions before and after overlap weighting and effective sample size**

**
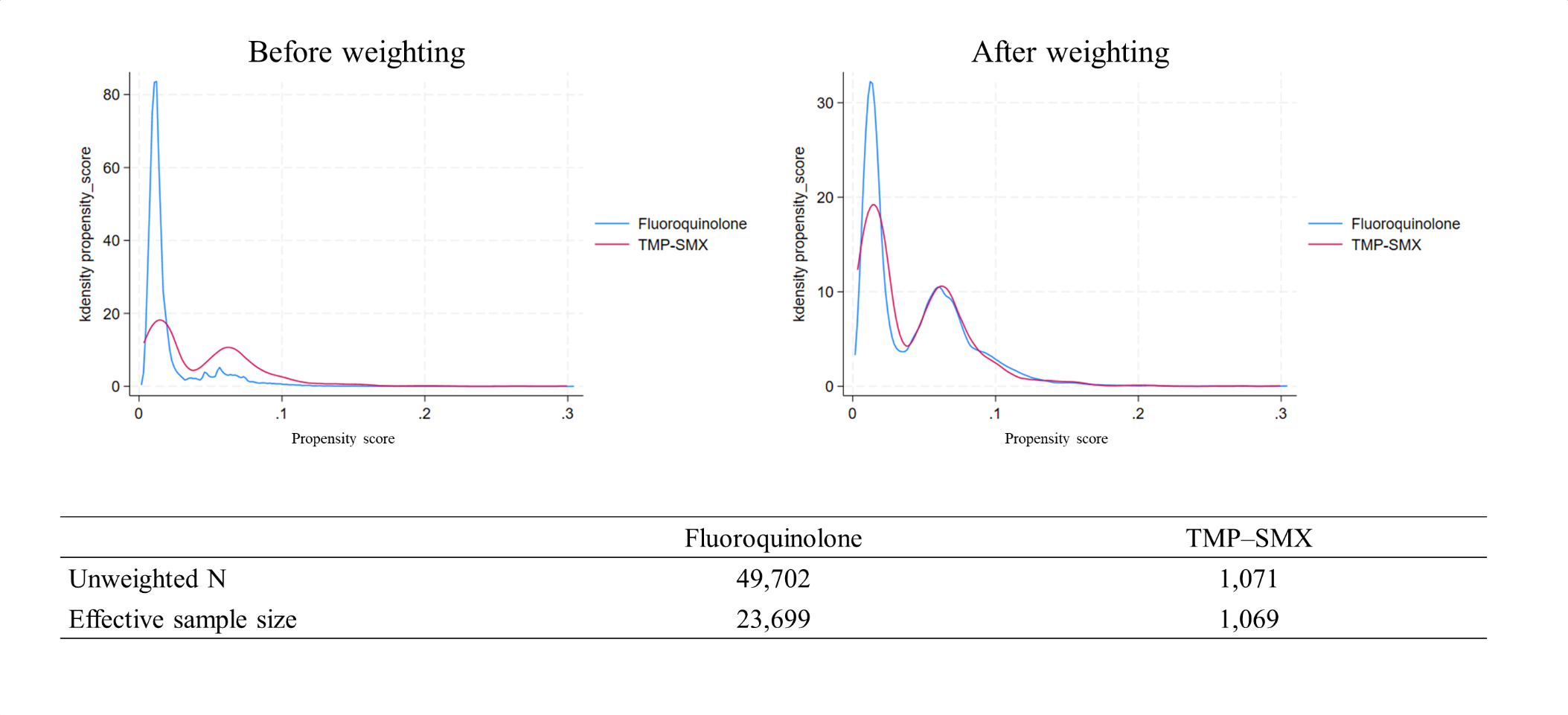
**

Propensity score distributions before and after overlap weighting for the TMP–SMX and fluoroquinolone groups. The table shows the unweighted sample size and effective sample size after overlap weighting.

**Supplementary Table 1**. **ICD-10 and ATC codes used for exclusion criteria**

| Diseases | ICD-10 codes |
| --- | --- |
| Urolithiasis | N20–N23 |
| Obstruction/hydronephrosis | N13, N32, N35 |
| Neurogenic bladder/ Voiding dysfunction | N31, R33, R39 |
| Congenital anomalies | Q60–Q64 |
| Diabetes mellitus | E10–E14 |
| Autoimmune disease | M05, M06, M30–M36 |
| HIV | B20–B24 |
| Chronic kidney disease | N18 |
| Malignancy | C00–C97, D00–D89 |
| Urinary tract infection | N10–N12, N30 |
| Pregnancy | O00–O99 |
| Drugs | ATC codes |
| Corticosteroids | H02AB |
| Immunosuppressants | L04 |
| Antineoplastic agents | L01 |
| Antibiotics | J01A, J01C, J01D, D01E, J01F, J01M, J01X |

ATC, Anatomical Therapeutic Chemical Classification System; ICD-10, International Classification of Diseases, 10th Revision

**Supplementary Table 2**. **ICD-10 and ATC codes used for covariates**

| Diseases | ICD-10 codes |
| --- | --- |
| Allergic rhinitis | J30 |
| Atopic dermatitis | L20 |
| Asthma | J45 |
| Urticaria | L50 |
| Hypertension | I10 |
| Dyslipidemia | E78 |
| Cerebrovascular disease | I60–I69 |
| Neuromuscular disorders | G10–G14, G20–G26, G70–G73 |
| Cardiovascular disease | I20–I25, I42, I50 |
| Liver disease | B16–B19, K70–K77 |
| Osteoporosis | M80–M82 |
| Osteoarthritis | M15–M19 |
| Gynecological disorders | N70–N77, N80–N98 |
| Dementia | F00–F03, G30 |
| Mood disorders | F30–F34, F38–F39 |
| Schizophrenia | F20–F25, F28–F29 |
| Drugs | ATC codes |
| Paracetamol | N02BE |
| NSAIDs | M01A |
| Herbal medicines | V03AX |
| Hormone replacement therapy | G03 |
| Diuretics | C03 |
| Psychotropic medications | N05AA, N05AD, N05AH, N05AX, N05CF, N05CD, N06AA, N06AB, N06AX |
| Proton pump inhibitors | A02BC |

ATC, Anatomical Therapeutic Chemical Classification System; ICD-10, International Classification of Diseases, 10th Revision

**Supplementary Table 3. ICD-10 and ATC codes for outcome definitions**

| Outcome category |  | Definition | ICD-codes | Additional requirements |
| --- | --- | --- | --- | --- |
| **Primary outcomes** |  |  |  |  |
| Anaphylaxis |  | Diagnosis of anaphylaxis with epinephrine administration | T78.2; T78.4 | Epinephrine use (ATC code: C01CA24) |
|  |  |  |  |  |
| Stevens–Johnson syndrome or toxic epidermal necrolysis |  | Diagnosis of Stevens–Johnson syndrome or toxic epidermal necrolysis | L51.1; L51.2 |  |
|  |  |  |  |  |
| Rash requiring pharmacotherapy |  | Diagnosis of rash/dermatitis requiring treatment | L27; L30; L50; L51.0; L51.8; L51.9; R21 | Oral/IV corticosteroid, or antihistamine (ATC code: R06A) + topical corticosteroid (ATC code: D07) |
|  |  |  |  |  |
| **Secondary outcomes** |  |  |  |  |
| Treatment failure |  |  |  |  |
|  | Upper urinary tract infection | Diagnosis of acute pyelonephritis | N10; N12 |  |
|  |  |  |  |  |
|  | Antibiotic modification | Change or addition of antibiotics within 7 days of the index date |  |  |
|  |  |  |  |  |
| All-cause hospitalization |  | Admission within 7 days of the index date |  |  |
|  |  |  |  |  |
| Other adverse events |  |  |  |  |
|  | Gastrointestinal symptoms | Nausea/vomiting, diarrhea, or noninfective gastroenteritis/colitis with treatment | R11; R19.7; K52 | Antiemetics (domperidone, metoclopramide), probiotics, or loperamide (ATC code: A03FA, A07FA, A07DA) |
|  | Electrolyte abnormalities | Diagnosis of hyponatremia or hyperkalemia | E87.1; E87.5 |  |
|  | Hypoglycemia | Diagnosis of hypoglycemia with glucose therapy | E16.0; E16.1; E16.2 | 50% glucose IV or oral glucose (ATC code: B05CX01) |
|  | Acute renal failure | Diagnosis of acute renal failure | N17 |  |
|  |  |  |  |  |
| **Serious arrhythmia or death** |  | Diagnosis of QT prolongation, ventricular tachycardia, or ventricular fibrillation/torsades de pointes | I45.8; I46; I47.2; I49.0 |  |

ATC, Anatomical Therapeutic Chemical Classification System; ICD-10, International Classification of Diseases, 10th Revision; IV, Intravenous.

**Supplementary Table 4. Comparison of outcomes between propensity score weighted groups in the sensitivity analyses**

|  | Fluoroquinolone | TMP-SMX | Risk difference | 95% confidence interval | *P* |
| --- | --- | --- | --- | --- | --- |
| **Patients with 3-Day Prescription (%)**  **[TMP-SMX (n = 544) vs. Fluoroquinolone (n = 7,904)]** |  |  |  |  |  |
| **Primary outcome** | 0.8 | 0.9 | 0.2 | −0.7 to 1.0 | 0.695 |
| **Secondary outcomes** |  |  |  |  |  |
| Treatment failure | 12.1 | 12.2 | 0.1 | −2.8 to 3.0 | 0.951 |
| All-cause hospitalization | 0.3 | 0.2 | −0.1 | −0.5 to 0.3 | 0.496 |
| Other adverse events | 0.2 | 0.2 | −0.1 | −0.4 to 0.3 | 0.773 |
|  |  |  |  |  |  |
| **Patients received TMP–SMX (160/800 mg twice daily) (%)**  **[TMP-SMX (n = 976) vs. Fluoroquinolone (n = 49,702)]** |  |  |  |  |  |
| **Primary outcome** | 0.7 | 0.9 | 0.2 | −0.4 to 0.8 | 0.452 |
| **Secondary outcomes** |  |  |  |  |  |
| Treatment failure | 11.4 | 10.0 | −1.4 | −3.3 to 0.6 | 0.167 |
| All-cause hospitalization | 0.2 | 0.2 | −0.0 | −0.3 to 0.3 | 0.846 |
| Other adverse events | 0.3 | 0.2 | −0.1 | −0.3 to 0.2 | 0.725 |
|  |  |  |  |  |  |
| **Patients without duplicate inclusion (%)**  **[TMP-SMX (n = 982) vs. Fluoroquinolone (n = 44,367)]** |  |  |  |  |  |
| **Primary outcome** | 0.7 | 0.9 | 0.2 | −0.4 to 0.8 | 0.474 |
| **Secondary outcomes** |  |  |  |  |  |
| Treatment failure | 11.4 | 11.1 | −0.3 | −2.3 to 1.7 | 0.770 |
| All-cause hospitalization | 0.2 | 0.3 | 0.1 | −0.3 to 0.4 | 0.672 |
| Other adverse events | 0.3 | 0.2 | −0.1 | −0.3 to 0.2 | 0.709 |
|  |  |  |  |  |  |
| **Patients diagnosed with anaphylaxis based on diagnostic codes alone (%)**  **[TMP-SMX (n = 1,071) vs. Fluoroquinolone (n = 49,702)]** |  |  |  |  |  |
| **Primary outcome** | 0.7 | 0.9 | 0.2 | −0.4 to 0.8 | 0.436 |
| Anaphylaxis | 0.0 | 0.0 | −0.0 | −0.0 to −0.0 | 0.001 |
| Stevens–Johnson syndrome/toxic epidermal necrolysis | 0.0 | 0.0 | N/A | N/A | N/A |
| Rash requiring pharmacotherapy | 0.7 | 0.9 | 0.2 | −0.3 to 0.8 | 0.410 |
|  |  |  |  |  |  |
| **Patients in IPTW-based ATU analysis (%)** |  |  |  |  |  |
| **Primary outcome** | 0.6 | 0.9 | 0.3 | −0.4 to 1.0 | 0.447 |
| **Secondary outcomes** |  |  |  |  |  |
| Treatment failure | 10.6 | 11.2 | 0.6 | −2.1 to 3.4 | 0.659 |
| All-cause hospitalization | 0.2 | 0.4 | 0.2 | −0.3 to 0.7 | 0.409 |
| Other adverse events | 0.2 | 0.3 | 0.1 | −0.5 to 0.6 | 0.844 |

ATU, Average Treatment effect on the Untreated; IPTW, Inverse Probability of Treatment Weight.
